# Criminal Abortion or Fœticide

**Published:** 1857-08

**Authors:** 


### CRIMINAL ABORTION OR FŒTICIDE.

We are glad to see that the attention of the profession is becoming fixed upon this subject, and we hope that it will not be allowed to rest until it is placed in its proper position in the scale of crime.

The laws upon this subject throughout, we believe, the entire world, show an utter ignorance or disregard of one of the most positive facts in Physiology, the effect of which has been to corrupt and degrade our species, and to destroy an almost incalculable amount of human health.

The product of conception should be looked upon as a living being, and its wanton destruction should be held as *murder*, whether it be with a view of concealing the evidence of a departure from virtue, or resorted to by the married for the purpose of getting rid of the cost or trouble of families.

While we have not had in the South the systematic pursuit of the infamous traffic alluded to, in an article in this number of our Journal, taken from the New Hampshire Journal of Medicine, yet even here, it is an evil of sufficient magnitude to demand the attention of all those who regard the welfare of the race.

